# Supplementary material for: High Angiotensin-Converting Enzyme and Low Carboxypeptidase N Serum Activity Correlate with Disease Severity in COVID-19 Patients
Source: J Pers Med. 2022 Mar 5;12(3):406. doi: 10.3390/jpm12030406 (PMC8949860; doi:10.3390/jpm12030406)
Supplement: Supplementary file 1 [file jpm-12-00406-s001.zip › jpm-1551673-supplementary.pdf]

High Angiotensin-Converting Enzyme and Low Carboxypeptidase N Serum Activity Correlate with Disease Severity in COVID-19 Patients

Supplementary materials:

Table S1. ACE2 and viral infection (assembled from [1, 2, 3] and references therein).

**ACE2 is functional receptor for SARS-CoV-2, required for host cell entry and subsequent viral replication**
ACE2 is mainly expressed by epithelial cells of lung, intestine, kidney, blood vessels, mucosa of oral cavity
Coronaviruses down-regulate ACE2 and thus its anti-inflammatory actions
Membrane-bound ACE2 is shed in COVID-19 infection, acute lung injury, myocardial infarction
Circulating ACE2 protects against influenza A virus induced acute lung injury
Turning off ACE2 gene causes severe lung damage in influenza-infected mice
Treating mice with human ACE2 dampens lung injury
Higher serum ACE2 levels in patients with better outcomes in influenza A induced lung injury

Table S2. ACE / ACE2-related factors relevant in COVID-19 progression (assembled from [1, 2, 3] and references therein).

	**ACE / ACE2**	**COVID-19**
Children	Children and adolescents have higher ACE and ACE2 levels in serum than adults	S*ymptomatic* COVID-19 in children and young adults uncommon (~1% < 19 y), disease progression less severe than in adults
	ACE2 levels and activity (urine, plasma, placenta) increase in pregnancy (protein can pass through the placenta)	Younger mice more resistant to infection, more severe disease in older animals
Gender	Estrogens participate in upregulation of ACE2 expression and activity, estradiol is modulator of ACE/ACE2 and AT1/AT2 receptor	No significant gender difference in disease progression in young patients
	Females have two ACE2 alleles and a greater range of phenotypes	Female mortality about half that of males, higher survival of females independent of older age
	Some ACE genotypes have twice the ACE activity than others	Men more likely to have more severe pathophysiological expression of the disease
	Males have only one X-linked ACE2 allele, thus, are more vulnerable to phenotypes causing impaired ACE2 regulation	
	Serum ACE2 levels lower in females	
	Higher tissue ACE2 levels in Asian females compared to males and other ethnic groups	
Age	ACE2 expression decreases with age	Death rate increases with age and co-morbidities (cardiovascular disease, diabetes, hypertension - “metabolic syndrome”)
Co-morbidities	Significant decrease in ACE2 expression in type II diabetes	More severe course with pre-existing cardiovascular disease
	Higher plasma ACE2 in advanced heart failure correlate with worsening clinical status	Higher levels of cardiac troponin correlate with more severe disease
	Serum ACE2 levels higher in hypertensive patients (up to 50% greater in males)	Serum Ang II levels elevated, correlate with viral load and lung injury
	Pre-existing hypertension correlates with greater frequency of ACE2 polymorphisms	Patients with severe disease present features consistent with unopposed RAS activity (higher blood pressure, lower serum potassium levels, high urinary potassium)

	High rates of vitamin D (negative endocrine RAS regulator) deficiency in patients requiring intensive care

References:

1. Arnold, R.G. COVID-19—Does this disease kill due to imbalance of the renin angiotensin system (RAS) caused by genetic and gender differences in the response to viral ACE2 attack? *Heart Lung Circ.* **2020**, *29*, 964–972.
2. Ciaglia, E.; Vecchione, C.; Puca, A.A. COVID-19 infection and circulating ACE2 Levels: Protective role in women and children. *Front. Pediatr*. **2020**, *8*, 206.
3. Skarstein Kolberg, E. ACE2, COVID19 and serum ACE as a possible biomarker to predict severity of disease. *J. Clin. Virol*. **2020**, *126*, 104350.
